# Retraction: Navigating board dynamics: Configuration analysis of corporate governance’s factors and their impact on bank performance

**DOI:** 10.1371/journal.pone.0334869

**Published:** 2025-10-23

**Authors:** The PLOS One Editors

The *PLOS One* Editors retract this article [1, corrected in 2] because it was identified as one of a series of submissions for which we have concerns about potential manipulation of the publication process, peer review integrity, and authorship. These concerns call into question the validity and provenance of the reported results. We regret that the issues were not identified prior to the article’s publication.

SHT, SG, and MZ did not agree with the retraction. MAS and HI either did not respond directly or could not be reached.
